# Immunogenicity and Protection Against Influenza H7N3 in Mice by Modified Vaccinia Virus Ankara Vectors Expressing Influenza Virus Hemagglutinin or Neuraminidase

**DOI:** 10.1038/s41598-018-23712-9

**Published:** 2018-03-29

**Authors:** Clement A. Meseda, Vajini Atukorale, Jackeline Soto, Maryna C. Eichelberger, Jin Gao, Wei Wang, Carol D. Weiss, Jerry P. Weir

**Affiliations:** 1 Laboratory of DNA Viruses, Center for Biologics Evaluations and Research, Food and Drug Administration, 10903 New Hampshire Ave, Silver Spring, MD 20993 USA; 2 Laboratory of Respiratory Viral Diseases, Center for Biologics Evaluations and Research, Food and Drug Administration, 10903 New Hampshire Ave, Silver Spring, MD 20993 USA; 3 Laboratory of Immunoregulation, Division of Viral Products, Center for Biologics Evaluations and Research, Food and Drug Administration, 10903 New Hampshire Ave, Silver Spring, MD 20993 USA

## Abstract

Influenza subtypes such as H7 have pandemic potential since they are able to infect humans with severe consequences, as evidenced by the ongoing H7N9 infections in China that began in 2013. The diversity of H7 viruses calls for a broadly cross-protective vaccine for protection. We describe the construction of recombinant modified vaccinia virus Ankara (MVA) vectors expressing the hemagglutinin (HA) or neuraminidase (NA) from three H7 viruses representing both Eurasian and North American H7 lineages – A/mallard/Netherlands/12/2000 (H7N3), A/Canada/rv444/2004 (H7N3), and A/Shanghai/02/2013 (H7N9). These vectors were evaluated for immunogenicity and protective efficacy against H7N3 virus in a murine model of intranasal challenge. High levels of H7-, N3-, and N9-specific antibodies, including neutralizing antibodies, were induced by the MVA-HA and MVA-NA vectors. Mice vaccinated with MVA vectors expressing any of the H7 antigens were protected, suggesting cross-protection among H7 viruses. In addition, MVA vectors expressing N3 but not N9 elicited protection against H7N3 virus challenge. Similar outcomes were obtained when immune sera from MVA vector-immunized mice were passively transferred to naïve mice prior to challenge with the H7N3 virus. The results support the further development of an MVA vector platform as a candidate vaccine for influenza strains with pandemic potential.

## Introduction

Modified vaccinia virus Ankara (MVA) vectors expressing influenza virus genes have shown promise as potential candidate vaccines, particularly for potential pandemic influenza viruses. The advantages of an MVA vector platform as a vaccine against influenza have been described previously^1,2^, and include the ability to elicit humoral and cellular immunity to expressed heterologous genes and demonstrated safety features due, at least in part, to the restricted replication in mammalian cells. Nevertheless, construction, manufacture, and evaluation of a new strain-specific MVA vector in response to an emerging pandemic influenza virus will require a considerable amount of time and may not be faster than a conventional vaccine response. On the other hand, if vectors are capable of generating significant cross-protective immune responses, candidate vaccine vector development and evaluation can be done prior to the emergence of a novel influenza virus, shortening the time needed for a successful vaccination response to the outbreak.

Pandemic preparedness for avian influenza has resulted in the construction and evaluation in various animal models of several MVA candidate vaccine vectors for H5 influenza viruses. Most of the characterized MVA vectors for avian H5 express the virus hemagglutinin (HA)^3–6^, and one MVA vector expressing the HA from the H5N1 A/Vietnam/1194/2004 virus has been assessed for safety and immunogenicity in a clinical trial in humans^7^. Evaluation of potential cross-protection has been encouraging. Not only have MVA vectors designed to enhance cross-reactivity, such as those expressing a mosaic H5 HA^4,8^ or multiple H5 HAs^6^, been shown to induce cross-protective responses, but relatively broad cross-protective responses have been demonstrated from MVA vectors expressing a single H5 HA^3,9,10^.

The emergence of the novel H7N9 virus in China, beginning in 2013^11,12^ but with yearly epidemics continuing^13^, is a vivid reminder that other avian subtypes of influenza also pose a serious public health threat and should be included in vaccine preparedness planning. Recently, an MVA vector expressing the H7 hemagglutinin was shown to protect ferrets against an H7N9 challenge^14^. But, as for H5 MVA vectors, more work is needed to define cross-reactivity and evaluate potential candidate H7 MVA vectors.

Here, we describe the construction of MVA vectors expressing the HA or neuraminidase (NA) from H7N3 and H7N9 viruses. MVA vectors were evaluated for their ability to elicit protective immunity in a mouse challenge model of H7N3. The results provide evidence for cross-protective responses to MVA-expressed HA from Eurasian and North American H7 lineages. In addition, the protective effect of MVA-expressed NA was demonstrated.

## Materials and Methods

### Cells and Viruses

The H7 viruses used in these studies were reassortant candidate vaccine viruses (http://www.who.int/influenza/vaccines/virus/en/). H7N3 A/mallard/Netherlands/12/2000 NIBRG-60 (A/mal/NL) was developed by the National Institute for Biological Standards and Control (NIBSC) (United Kingdom); H7N3 A/Canada/rv444/2004 (A/Can) was developed at the St. Jude Children’s Research Hospital (Memphis, Tennessee); H7N9 A/Shanghai/02/2013 (IDCDC-RG32A) (A/Shang) was developed at the Centers for Disease Control and Prevention (Atlanta, Georgia). Influenza viruses were propagated in 9-day-old specific pathogen-free embryonated chicken eggs. For the isolation of genomic RNA for the cloning of viral genes, the three H7 viruses were propagated in Madin-Darby canine kidney (MDCK) cells. The modified vaccinia virus Ankara (MVA) was obtained from Dr. Bernard Moss, NIAID/NIH, and was propagated in DF-1 cells (ATCC **#** CRL-12203). The purification of MVA and recombinant MVA vectors from infected DF-1 cells by ultracentrifugation through a 36% sucrose cushion was performed as previously described^15,16^. All experiments, including animal experiments, were conducted under biosafety level 2 plus (BSL-2+) laboratory conditions.

### Construction of MVA Vectors Expressing Influenza A/H7 HA or NA Genes

MDCK cells in 6-well tissue culture plates were infected with influenza A virus (A/Can, A/mal/NL, or A/Shang) at a multiplicity of infection (MOI) of 1.0. After 24 hours, cells were scrapped and harvested by low speed centrifugation at 1000 rpm for 10 min at 4 °C. Total RNA was isolated from each cell pellet using the RNeasy mini kit and protocol described by the manufacturer (Qiagen Inc., Germantown, MD). Viral cDNAs were synthesized from total RNAs using the Omniscript reverse transcription kit (Qiagen) and the 12-mer Uni-12 influenza universal primer^17^. Using the cDNAs as templates, specific primer pairs (Table 1) were used for the amplification of the HA and NA genes of A/Can, A/mal/NL, and A/Shang by polymerase chain reaction (PCR), using the high fidelity Phusion-HF DNA polymerase (New England Biolabs, Ipswich, MA).

**Table 1:** Oligonucleotide primers used in the amplification of HA and NA genes of A/H7 viruses.

Name	*Oligonucleotide sequence	Description
CM-13	AGCAAAAGCAGG	Uni-12 influenza universal primer^17^
**HA genes**		
nCM-1	**CACC**ATGAACACACAGATTCTGGCTTTCA	Forward for A/Can HA
nCM-2	GCTCAAATACAGATGGTGCAGCGCAT	Reverse for A/Can HA
nCM-3	**CACC**ATGAACACCCAGATCCTGGTGTT	Forward for A/mal/NL HA
nCM-4	GCTCAGATACAGATGGTGCAGCGCAT	Reverse for A/mal/NL HA
CM-20	**CACC**ATGAACACTCAAATCCTGGTA	Forward for A/Shang HA
CM-21	CTTATATACAAATAGTGCACCGCAT	Reverse for A/Shang HA
**NA genes**		
CM-54	**CACC**ATGAATCCGAATCAGAAGATAATAACA	Forward for A/Can NA
CM-55	CTATTACTTGGGCATAAACCCAATGTT	Reverse for A/Can NA
CM-24	**CACC**ATGAATCCAAATCAGAAGATAATAACAA	Forward for A/mal/NL NA
CM-25	GTTACTTGGGCATAAACCCAATGTT	Reverse for A/mal/NL NA
CM-22	**CACC**ATGAATCCAAATCAGAAGATTCTA	Forward for A/Shang NA
CM-23	GGTTAGAGGAAGTACTCTATTTTAGCCCCAT	Reverse for A/Shang NA

*The sequence CACC (in bold typeface) was added to the 5′ end of each forward primer to facilitate directional insertion of each PCR amplicon into the pENTR-D-TOPO vector. All oligonucleotide primers were synthesized by the Facility for Biotechnology Resources, CBER/FDA.

Each PCR product was ligated with pENTR-D-TOPO vector (ThermoFisher Scientific, Rockford, IL) to generate entry clones. The authenticity of the HA and NA nucleotide sequences in the pENTR-D-TOPO vector was confirmed by nucleotide sequencing. Each entry clone was recombined with plasmid pLW44attR^18^ by LR recombination (ThermoFisher Scientific) to generate pLW44-HA or pLW44-NA shuttle vectors. In making recombinant MVA, monolayers of DF-1 cells were infected with MVA at an MOI of 0.1 for 1 hour, followed by transfection with pLW44-HA or pLW44-NA. Recombinant MVA expressing HA (MVA-HA) or NA (MVA-NA) were obtained from the infected/transfected cells by serial plaque purification (3 to 6 times) of recombinant plaques on DF-1 cells. Genetic stability of recombinant vectors was confirmed by plaque assay and the absence of parental MVA in the purified recombinant vectors was confirmed by PCR assay. Expression of authentic HAs and NAs by the recombinant MVA vectors was confirmed by Western blotting. For downstream use in animal inoculation, recombinant MVA-HA and MVA-NA were further pelleted on 36% sucrose cushion as previously described^15,16^.

### Animals and Immunizations

All immunizations and blood draws were performed in accordance with an animal protocol approved by the FDA White Oak Consolidated Animal Program. Six to eight week-old male BALB/cByJ mice were purchased from the Jackson Laboratory (Bar Harbor, Maine). For immunization, MVA-HA or MVA-NA vectors, as well as MVA control, were diluted to the appropriate dose in plaque-forming units (PFU) (10^2^ PFU–10^8^ PFU per dose in 50–100 µL) in sterile, endotoxin-free phosphate-buffered saline (PBS). Mice were inoculated subcutaneously at the base of the tail, and evaluated daily for up to 7 days post-vaccination. Blood samples were obtained from mice three weeks post-vaccination.

### Antibody ELISA

An enzyme-linked immunosorbent assay (ELISA) for the detection of specific immunoglobulin G (IgG) responses to H7 was developed and optimized using recombinant H7 protein of A/Anhui/1/2013 (H7N9) produced in baculovirus (Cat. #NR-45118; BEI Resources, Manassas, Virginia). Immulon 2HB plates were coated with the recombinant H7 protein at 1 µg/mL in PBS, and stored at 4 °C overnight. Excess coating solution was removed from plates, and blocking buffer (PBS containing 10% fetal bovine serum [FBS]) was added to wells, and plates were incubated for two hours at 37 °C. Plates were washed in wash buffer (PBS containing 0.05% Tween-20) and incubated with serial two-fold dilutions of each test serum sample diluted in wash buffer containing 10% FBS as the diluent. After incubation for 1.5 to 2 hours, plates were washed and incubated with a 1:5,000 dilution of goat anti-mouse IgG, horseradish peroxidase (HRP) conjugate (SouthernBiotech, Birmingham, AL) for 1 hour. Plates were washed and incubated with the HRP substrate 2,2′-Azinobis [3-ethylbenzothiazoline-6-sulfonic acid] hydrogen peroxide (KPL, Gaithersburg, Maryland) for 30 minutes. After stopping the HRP/substrate reaction with 1% sodium dodecyl sulfate (SDS), the OD_405_ was read on a VersaMax microplate reader and the data were analyzed with Softmax Pro, using a column of eight wells on each plate in which the diluent was added as test sample, as the blank. The IgG titer of each test serum was defined as the reciprocal of the highest serum dilution that gave a mean OD_405_ value of ≥0.05, as previously described^19^.

Similar ELISAs were set-up for the detection of the IgG response to influenza NA (N3 and N9) in test serum samples, except that plates were coated with recombinant N3 protein of A/Canada/rv444/2004 (H7N3) (cat.# FR-478; Influenza Reagent Resource, Manassas, Virginia) or recombinant N9 protein of A/Shanghai/2/2013 (H7N9) (cat.# NR-44364, BEI Resources) for the quantitation of N3-specific and N9-specific antibodies, respectively. In calculating mean antibody titers, a serum sample with no detectable IgG was assigned a titer of 10 (one 2-fold dilution below the initial serum dilution used in the assay).

### Cytokine Assays

Groups of naïve mice (3 mice per group) were inoculated with 10^8^ PFU of recombinant MVA-HA vectors or with parental MVA, via the subcutaneous route. A control group was left as untreated (naïve). On day 7 post-inoculation, all mice were sacrificed, dissected, and spleen isolated from each mouse as previously described^19^. Lymphocytes were prepared from each mouse, cultured in Roswell Park Memorial Institute 1640 (RPMI-1640) medium at 10^6^ cells/well in 24-well tissue culture plates, and stimulated *in vitro* by live virus infection with candidate vaccine viruses A/Can or A/mal/NL or A/Shang. Culture supernatants were collected after 72 hours incubation, clarified by centrifugation, and tested for secreted IFN-γ, as previously described^19^.

### Mouse Intranasal Challenge

The intranasal challenge route was used for the assessment of protective immune responses in mice. Mice were weighed and anesthetized by intraperitoneal injection of ketamine/xylazine (0.5 mg/0.1 mg per gram body weight), and challenged with 5 to 10 50% lethal dose (LD_50_) of A/mallard/Netherlands/12/2000 (H7N3) which proved to be more lethal than A/Can and A/Shang vaccine viruses, as determined in preliminary LD_50_ experiments. Challenge virus at the appropriate dose was re-suspended in sterile PBS, and each anesthetized mouse received 10 µL of the suspension per nostril. Following challenge, mice were evaluated and weighed daily for two weeks. The study endpoint was a 25% decrease in body weight from the weight on the day of challenge. A mouse that reached this study endpoint was sacrificed and considered to have succumbed to infection.

### Passive Antiserum Transfer

Hyperimmune antisera were prepared by immunizing groups of mice (5 to 10 mice per group) with 10^8^ PFU of MVA-HA or MVA-NA or parental MVA, by prime/boost at an interval of three weeks. Immune serum was obtained from each group three weeks after each immunization. Serum samples were pooled by treatment group and tested for the neutralization of lentiviral pseudoviruses expressing the HA of H7 viruses and for neuraminidase inhibition (NI). For passive transfer, each antiserum was diluted 1:1.5 in sterile PBS, and inoculated into groups of naïve mice (5 mice per group) with 0.5 mL per mouse (i.e., 0.33 mL of antiserum), via the intraperitoneal (i.p.) route. After 6 hours, mice were weighed, anesthetized, and challenged with 5 LD_50_ of A/mallard/Netherlands/12/2000 (H7N3) as described above, and evaluated over two weeks.

### Neuraminidase Inhibition Assay

The enzyme-linked lectin assay (ELLA) was used for the detection of NA-inhibiting antibodies in immune antisera as previously described^20^. Since the recombinant MVA expressed NA alone, the homologous H7N3 influenza virus preparations could be used as the source of NA in the ELLA without any concern of interference by HA-specific antibodies. An H6N9 reassortant virus generated by reverse genetics as previously described^21^ was used to measure N9-specific antibody titers.

### HA-pseudovirus Production and Neutralization

H7 HA from A/mallard/Netherlands/12/2000, A/Shanghai/2/2013 and A/Canada/rv444/2004 were used to construct retroviral pseudoviruses. HA-pseudoviruses were produced in 293 T cells essentially as described previously^22,23^. HA-pseudovirus neutralization was tested as described^24^. The antibody dilution causing a 95% reduction of pseudovirus-expressed luciferase compared to control was used as the neutralization endpoint titer (IC_95_-neutralizing antibody titer), and was calculated with nonlinear regression using Prism software (GraphPad, La Jolla, CA). Data reported were from at least two independent experiments, with each sample run in duplicate.

### Statistical Analysis

Differences in antibody responses to HA or NA between treatment groups inoculated with different doses of MVA-HA or MVA-NA were compared using a statistical t-test of the log10-transformed IgG titers or the Mann-Whitney rank sum test where an initial test of normality failed. In all cases a p-value < 0.05 was considered statistically significant.

### Data availability

All data generated or analyzed during this study are included in the text, tables, and figures.

## Results

### Construction of modified vaccinia virus Ankara (MVA) vectors expressing hemagglutinin (HA) and neuraminidase (NA) genes from influenza H7 viruses

Recombinant MVA vectors were constructed expressing the HA or NA from two H7N3 viruses and one H7N9 virus. One H7N3 virus, A/mallard/Netherlands/12/2000 (A/mal/NL) is from the group II H7 Eurasian lineage^25^; the second H7N3, A/Canada/RV444/2004 (A/Can), is a North American lineage H7 influenza isolate. The H7N9 virus, A/Shanghai/2/2013 (A/Shang), is an H7 virus of Eurasian origin (group I lineage) from the recent 2013 H7N9 outbreak in China. The construction and purification of the recombinant viruses are described in Materials and Methods.

**Figure 1:**
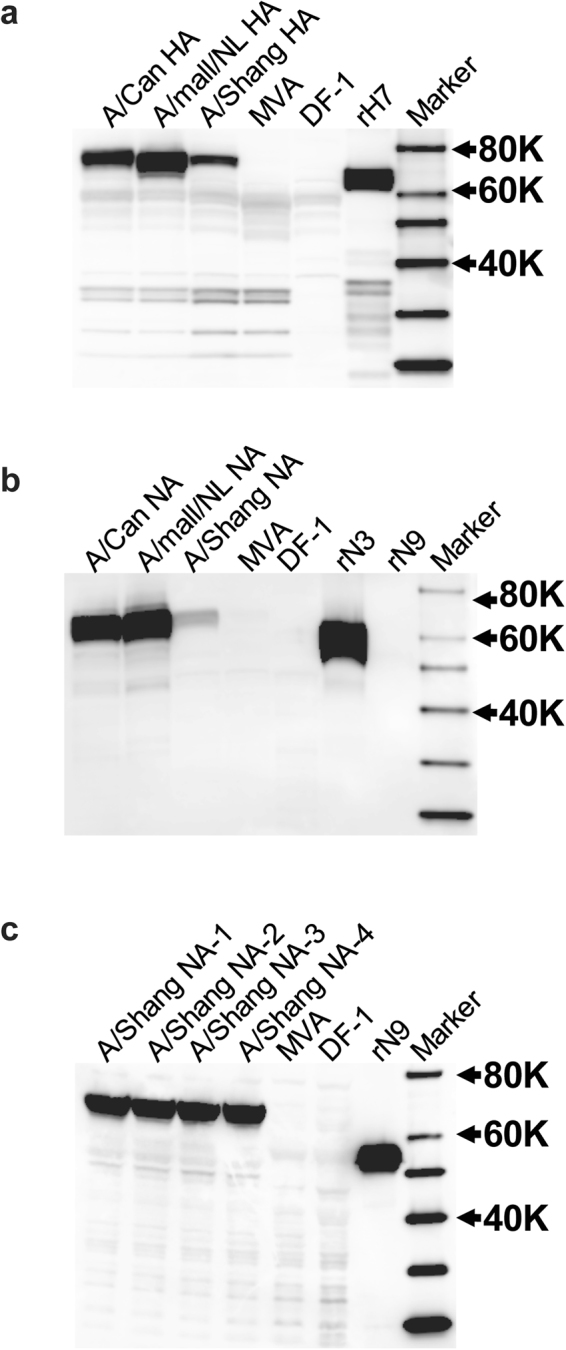
Western blot analysis of recombinant MVA vectors expressing influenza HA or NA. CEF cells were infected with MVA-HA or MVA-NA vectors at an MOI of 1.0. Infected cells were harvested after 48 hours and the cell lysates resolved by SDS-PAGE (4–12% gels). Proteins were transferred onto nitrocellulose membranes and probed with a polyclonal rabbit anti-H7 (A/Shanghai/02/2013 (H7N9)) for the detection of H7 (**a**), a polyclonal goat anti-N3 (A/turkey/England/1963 (H7N3)) for the detection of N3 (**b**), or with a polyclonal goat anti-N9 (A/tern/Australia/G70C/1975 (H11N9)) for the detection of N9 (**c**). HRP-conjugated anti-rabbit or anti-goat antibodies were used as detection antibodies, respectively. After the addition of Supersignal West Dura Luminol HRP substrate, chemiluminenscent images were captured under a Fujifilm Intelligent Dark Box, using the Image Reader LAS-3000 software. The full Western blot images for Fig. 1 are provided in the Supplementary Info File.

Recombinant proteins expressed by the MVA vectors were characterized by Western blotting. Lysates of cells infected with the recombinant viruses were resolved by SDS-polyacrylamide gel electrophoresis (SDS-PAGE) and the blotted proteins on the membrane matrix were detected with antibodies specific to H7 or N3 or N9 (Fig. 1). The H7 of A/Can, A/mal/NL, and A/Shang were detected as the uncleaved HA0 form, all migrating at about the same size of 75–80 kDa (Fig. 1a). By contrast, lysates of DF-1 cells or MVA-infected cells had no detectable band corresponding to HA. A baculovirus recombinant HA (H7N9 A/Anhui/1/2013) included as a positive control was also detected in the HA0 form, but migrated at about 70–75 kDa, possibly due to differences in the extent of glycosylation between cells of avian and insect origins. Blots of lysates from MVA-NA infected cells were probed with an N3-specific antibody (Fig. 1b). All three NAs were detectable as approximately 60–65 kDa bands. However, the reactivity with the N9 of A/Shang was much weaker than N3. Neither the MVA-infected cells nor uninfected DF-1 cell control had a detectable band corresponding to NA. To further confirm the authenticity of N9 expression, a blot containing four independent lysates of MVA-NA (A/Shang; N9) infected cells along with MVA-infected cell lysate and uninfected cell lysate, and a baculovirus/insect cell-produced recombinant N9 protein, was probed with an N9-specific antibody. There were no bands detected in the MVA and uninfected cell lanes. A 60–65 kDa band corresponding to N9 was detectable in the two N9 lanes (A/Shang NA-1 and A/Shang NA-2 (Fig. 1c). Similarly, an approximately 55–58 kDa band was detected in the lane containing the insect cell-derived recombinant N9. Thus, the detection of the HA and NA bands indicate that the recombinant MVA vectors express the expected H7, N3, and N9 proteins.

### Immunogenicity and protective effect of MVA vectors expressing HA and NA from influenza H7 viruses

**Figure 2:**
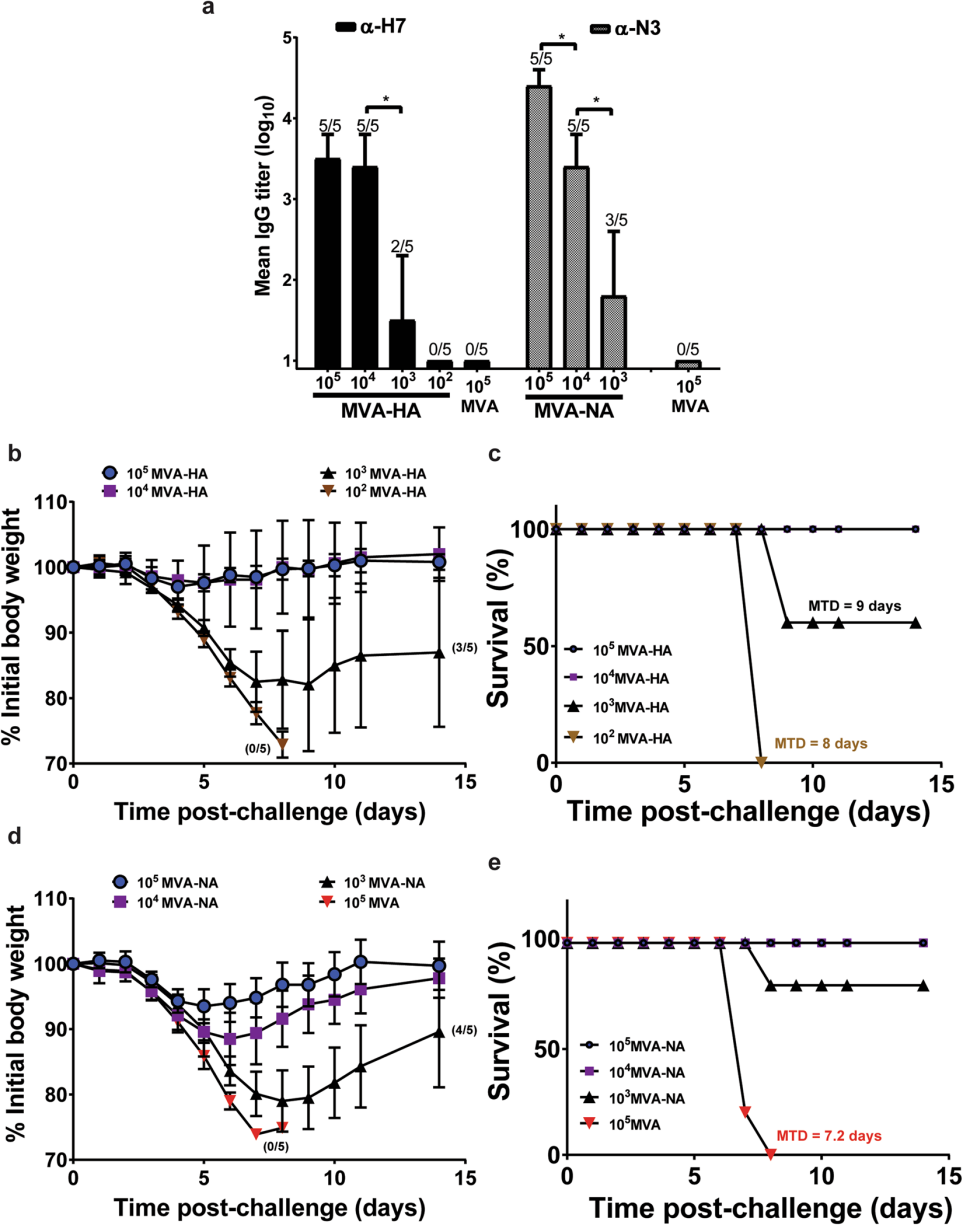
Dose response to MVA-HA and MVA-NA. Groups of mice (5/group) were vaccinated subcutaneously with a range of doses of 10^2^ to 10^5^ PFU of MVA vector expressing the HA of A/mal/NL or 10^3^ to 10^5^ PFU of MVA expressing the NA of A/mal/NL. Sera were obtained 3 weeks after vaccination and tested for H7-specific IgG or N3-specific IgG (**a**). At three weeks post-vaccination, each mouse was challenged with 5 LD_50_ (7.9 × 10^4^ PFU) of influenza A/mallard/Netherlands/12/2000 (H7N3) via the intranasal route. Mice were weighed daily for two weeks. Weight loss curves for the MVA-HA treatment cohort and MVA-NA treatment cohort are shown in (**b**,**d**). Survival curves for the MVA-HA treatment cohort and MVA-NA treatment cohort are shown in (**c**,**e**). An asterisk denotes a significant difference between indicated groups (*p* < 0.05).

To evaluate the immunogenicity and protective effect of recombinant vectors expressing HA and NA, a lethal challenge model was established using A/mal/NL. This virus, which can be handled under BSL-2+ conditions, resulted in morbidity, as measured by weight loss and mortality, with an LD_50_ of 1.6 × 10^4^ PFU when inoculated into mice by the intranasal route. In order to determine whether MVA vectors expressing the A/mal/NL HA or NA would elicit protective responses against the A/mallard/Netherlands/12/2000 challenge virus, dose-ranging studies of the recombinant vectors were performed. Mice were vaccinated with a range of doses of the MVA vector expressing the A/mal/NL HA (10^2^–10^5^ PFU) or NA (10^3^–10^5^ PFU). A control group was vaccinated with 10^5^ PFU of MVA. Three weeks post-vaccination, serum samples obtained from the animals were tested for H7-specific IgG (for the MVA-HA treatment cohort) or N3-specific IgG (for the MVA-NA treatment cohort) by ELISA. Mice in the MVA control group had no quantifiable H7-specific or N3-specific IgGs (Fig. 2a). Similarly, none of the mice in the 10^2^ PFU MVA-HA treatment group was seropositive for H7-specific IgG. By contrast, all mice vaccinated with ≥10^4^ PFU of MVA-HA were seropositive for H7 IgG, with mean log_10_ IgG titers ± standard deviation (SD) of 3.5 ± 0.3 and 3.4 ± 0.4 for the 10^5^ and 10^4^ PFU MVA-HA groups, respectively (Fig. 2a); these IgG titers were not significantly different. Similarly, all mice vaccinated with ≥10^4^ PFU of MVA-NA were seropositive for N3-specific IgG, with log_10_ IgG titers ± SD of 4.4 ± 0.2 and 3.4 ± 0.4, for the 10^5^ and 10^4^ PFU MVA-NA groups, respectively; the IgG titers in these two groups were significantly different. In the 10^3^ PFU immunization groups, 2/5 (mean titer = 1.5 ± 0.8) and 3/5 (mean titer = 1.8 ± 0.8) in the MVA-HA and MVA-NA, respectively, had measurable levels of specific IgG; these IgG titers were significantly lower than those in the 10^4^ PFU groups.

All mice were challenged at 3-weeks post-immunization with 5 LD_50_ of A/mal/NL; disease progression post-challenge is shown in Fig. 2b,d; survival curves are shown in Fig. 2c,e. As reflected by the antibody responses, all mice in the MVA control group and the 10^2^ PFU MVA-HA group lost weight dramatically and succumbed to infection, with a mean time-to-death (MTD) of 7.2 and 8 days, respectively. All mice vaccinated with ≥10^4^ PFU of MVA-HA or MVA-NA survived challenge, with the MVA-HA treatment cohort losing less weight than the MVA-NA treatment cohort. This set of data indicates that recombinant H7 or N3 expressed from MVA vectors elicit protective responses against the homologous H7N3 virus in mice, and shows that doses ≥10^4^ PFU are protective in this model.

### Effect of co-administration of A/mal/NL MVA-HA and MVA-NA against A/mallard/Netherlands/12/2000 challenge

Since both HA- and NA-expressing MVA vectors appeared protective against H7N3 challenge, we tested whether immunization with combinations of MVA-HA and MVA-NA would be more effective than either MVA vector alone in providing a protective response. For this purpose, immunization with sub-optimal doses of 10^3^ PFU and 10^4^ PFU of the MVA vectors expressing the HA or NA of A/mal/NL were selected, since our data above indicated that a dose of 10^3^ PFU of either MVA-HA or MVA-NA provided only partial protection against A/mal/NL challenge and weight loss was also observed at the 10^4^ PFU dose. Four groups of mice (5 per group) were vaccinated with either MVA-HA only (10^3^ PFU and 10^4^ PFU) or MVA-NA only (10^3^ PFU and 10^4^ PFU). Two other groups were vaccinated with a mixture of 10^3^ PFU MVA-HA and 10^3^ MVA-NA or a mixture of 10^4^ PFU MVA-HA and 10^4^ MVA-NA. For the groups that received 10^3^ or 10^4^ PFU of MVA-HA or MVA-NA only, the equivalent dose of MVA was mixed with each dosage in order to normalize for the MVA background among all six treatment groups. One group of mice was vaccinated with 10^6^ PFU of MVA-HA as a positive control, and one group was vaccinated with 10^6^ PFU of MVA as a negative control. Three weeks post-vaccination, sera were obtained from all mice and tested for H7-specific IgG and N3-specific IgG by ELISA (Table 2).

**Table 2:** HA and NA-specific IgG antibody titers in MVA-vector immunized mice.

**Immunization**	**Mean IgG Titer (log**_**10**_ **)**		**Protection**
	H7 (#positive)	N3 (#positive)	
10^3^ MVA-HA	1.4 ± 0.8 (1/5)	N.D.^a^	No
10^3^ MVA-NA	N.D.	2.4 ± 1.3 (3/5)	No
10^3^ MVA-HA 10^3^ MVA-NA	1.0 (0/5)^b^	1.8 ± 0.8 (3/5)	No
10^4^ MVA-HA	2.4 ± 1.3 (3/5)	N.D	Yes
10^4^ MVA-NA	N.D.	3.8 ± 0.3 (5/5)	Yes
10^4^ MVA-HA 10^4^ MVA-NA	2.2 ± 1.1 (3/5)	3.5 ± 0.6 (5/5)	Yes
10^6^ MVA-HA	4.2 ± 0.1 (5/5)	N.D.	Yes
10^6^ MVA	1.0 (0/5)	1.0 (0/5)	No

^a^N.D. – Not done.

^b^For titer calculations, test samples with a titer <20 (below quantitation) were assigned a value of 10.

In the 10^3^ and 10^4^ MVA-HA only treatment groups, 1/5 and 3/5 mice, respectively, had detectable H7 IgG. Among the MVA-NA only treated groups, 3/5 and 5/5 mice in the 10^3^ and 10^4^ PFU groups, respectively, had detectable N3-specific IgG. Among the MVA-HA/MVA-NA mixed vaccination cohorts, none of the mice in the 10^3^ PFU group had detectable H7 IgG while 3/5 mice had detectable anti-N3 IgG. There were no statistical differences in the antibody response to HA or NA between groups that received a single antigen (MVA-HA or MVA-NA) and those vaccinated with a combination of MVA-HA/MVA-NA. In the 10^4^ group, 3/5 mice had detectable H7 IgG titers and 5/5 mice had anti-N3 IgG. Again, there were no statistical difference between the single antigen groups and the antigen combination group. H7-specific and N3-specific IgGs were not detected in the sera of mice in the MVA control group, but all mice in the high-dose 10^6^ MVA-HA group had high titers of anti-H7 IgG. Data from these experiments confirm a dose response to vaccination with the MVA vectors, with antibody titers increasing with dose escalation. Further, co-administration of vectors expressing H7 and N3 did not appear to interfere with the IgG response to each antigen.

**Figure 3:**
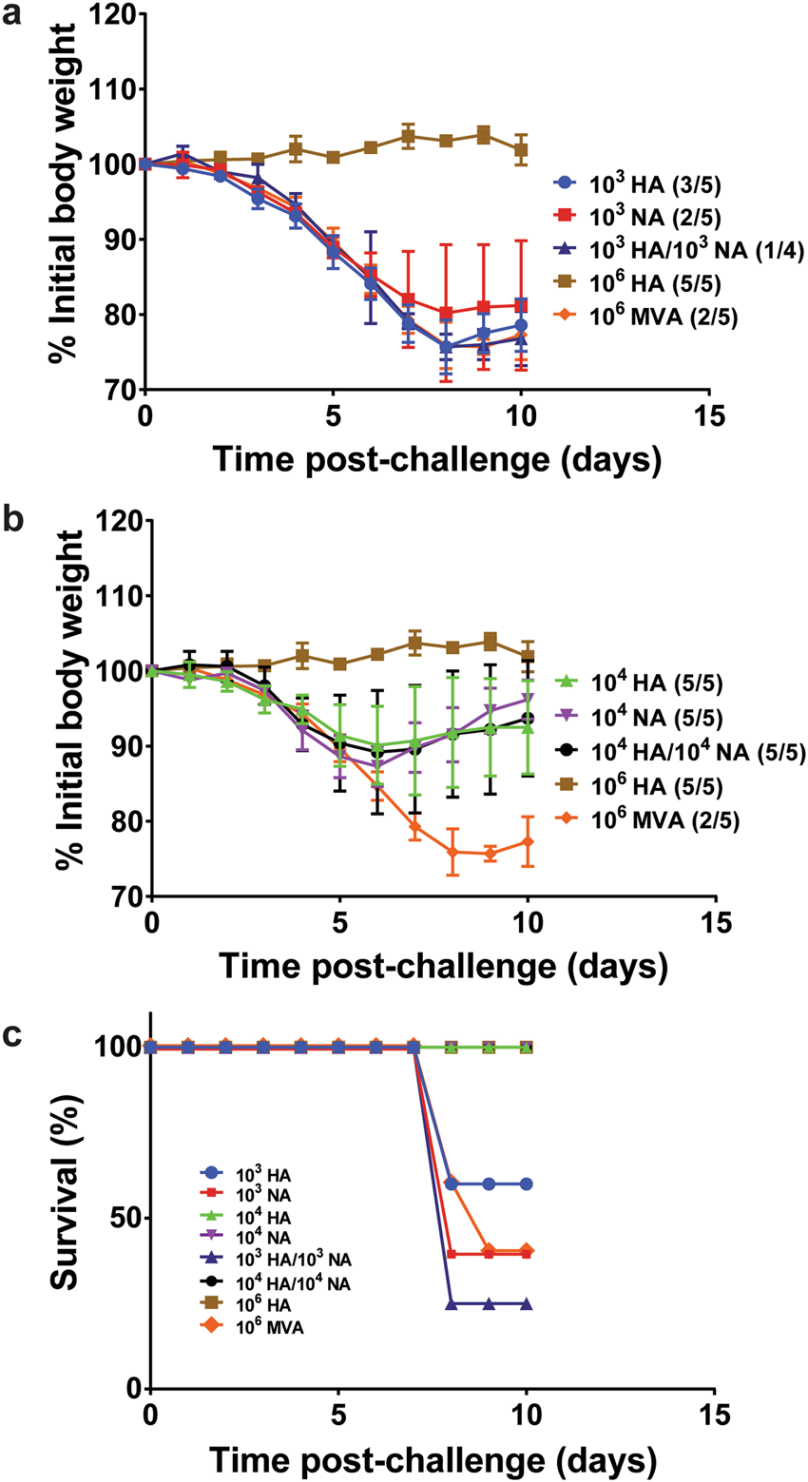
Protective effect of combinations of MVA-HA and MVA-NA. Groups of mice (5 per group) were vaccinated with MVA-HA or MVA-NA (HA and NA from A/mal/NL) individually or in combinations of MVA-HA and MVA-NA. A positive control group was vaccinated with 10^6^ PFU MVA-HA, and a negative control group was vaccinated with 10^6^ PFU MVA. Mice were challenged with 5 LD_50_ of A/mal/NL, and the weight loss curves are shown for the 10^3^ MVA vector treatment groups (**a**) and 10^4^ vector treatment groups (**b**). Weight loss curves for the positive and negative control groups are shown in both graphs. The number of surviving mice in each group is indicated in parentheses, and the survival curves for all groups are shown in (**c**).

In order to assess the effect of co-expression of H7 and N3 on the protection of mice from influenza, mice in this study were challenged with 5 LD_50_ of A/mal/NL, and evaluated daily. Figure 3a,b shows influenza morbidity post-challenge as measured by weight loss or gain; Fig. 3c shows the survival curves for each group. None of the mice in the 10^6^ PFU MVA-HA positive control group lost weight, and all survived virus challenge. In the MVA (negative control) group, 3/5 mice succumbed to infection, and although two mice survived, their respective peak weight loss (23% and 23.5%, on study day-9) was close to the study endpoint of 25%. At low-dose vector immunization, 3/5 mice in the 10^3^ PFU MVA-HA and 2/5 of those in the 10^3^ PFU MVA-NA survived challenge and maximum weight loss of the survivors in these groups was near the study endpoint. Adding 10^3^ MVA-HA and 10^3^MVA-NA did not confer any obvious protective advantage against challenge. One mouse in the combined 10^3^ PFU MVA-HA/10^3^ MVA-NA treatment group was found dead a day after challenge; the death was considered unrelated to the virus challenge. Of the remaining four evaluable mice in the group, three succumbed to infection with a mean time-to-death of 8 days.

In a pattern consistent with our previous observations, all mice vaccinated with 10^4^ PFU of MVA-HA only or 10^4^ MVA-NA only survived challenge, albeit with significant peak mean weight losses of 9.9% (on day 6) and 12.7% (on day 6), respectively. All mice vaccinated with a mixture of 10^4^ PFU MVA-HA/10^4^ MVA-NA also survived, but the peak mean weight loss (10.8% on day 6) was similar to those of the 10^4^ PFU MVA-HA only or MVA-NA only treatment groups, again suggesting a lack of additive effect against challenge. The results of this virus challenge study corroborated our previous observation that animals vaccinated with ≥10^4^ PFU of MVA vectors expressing either influenza virus H7 or N3 were protected from lethal challenge with the H7N3 homologous virus. Although, immunization with a combination of MVA-HA and MVA-NA vectors did not seem to enhance protection in this mouse model, there appeared to be no inhibitory effects of combining the two vectors.

### Protection against H7N3 A/mallard/Netherlands/12/2000 challenge from MVA vectors expressing HA or NA from heterologous H7 viruses

**Figure 4:**
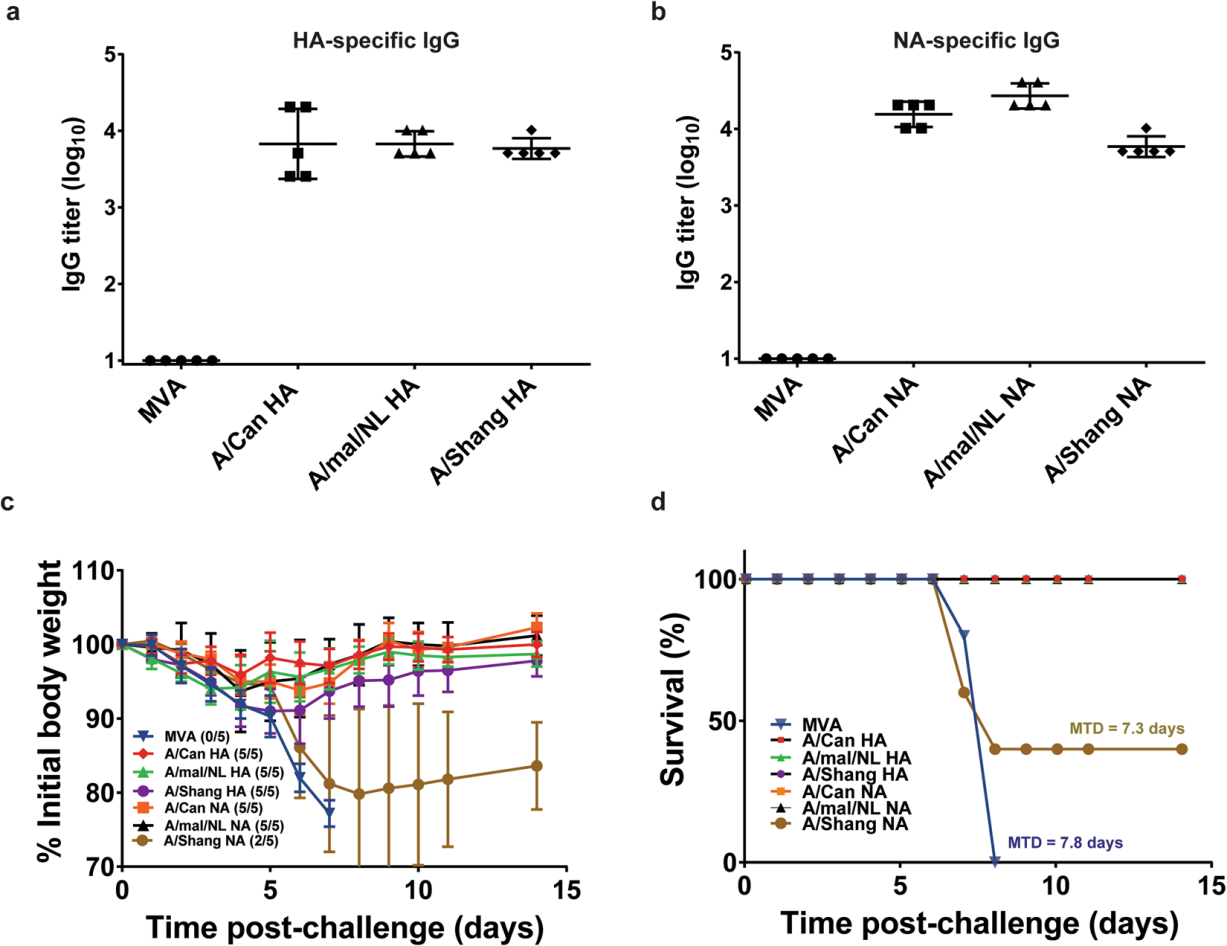
Immunogenicity and protective effectiveness of MVA-HA or MVA-NA. Groups of mice (5 per group) were vaccinated with 10^7^ PFU of recombinant MVA-HA or MVA-NA vectors. A control group was vaccinated with 10^7^ PFU of MVA. After 3 weeks, sera were collected from mice and tested for H7-specific IgG in an antigen capture ELISA, using purified recombinant H7 protein (**a**). NA-specific IgG was assayed using purified recombinant N3 protein or purified recombinant N9 protein (**b**). Data show the individual IgG titers. The horizontal bar indicates the mean IgG titer and error bars represent standard deviation. All mice were challenged 3 weeks post-vaccination with 10 LD_50_s of influenza A/mallard/Netherlands/12/2000 (H7N3) via the intranasal route. Weight loss is shown in (**c**), with the number of surviving mice in each group indicated in parentheses. Survival curves for the different treatment groups are shown in (**d**).

In order to determine whether MVA vectors expressing the HA or NA of heterologous H7 influenza viruses can provide protection against A/mal/NL H7N3 challenge, mice were vaccinated with 10^7^ PFU of MVA-HA or MVA-NA (HA and NA derived from H7N3 A/Can, H7N3 A/mal/NL and H7N9 A/Shang). Three weeks post-vaccination, sera were obtained from mice and tested for H7- or N3 or N9-specific IgGs by ELISA. No mice inoculated with the empty MVA vector had detectable IgG against HA or NA (Fig. 4a,b), but all mice inoculated with MVA-HA or MVA-NA had detectable levels of antigen-specific IgGs. Because of the relatively high dose of MVA vector used for immunization, mice in this experiment were challenged with 10 LD_50_ of influenza A/mal/NL. By 7 to 8 days post-challenge, all mice in the MVA control group reached the study endpoint of 25% weight loss (Fig. 4c,d). By contrast, all mice vaccinated with MVA vectors expressing HA (H7) survived, irrespective of the virus source of the H7, suggesting cross-protection was provided by MVA-H7 expressing vectors.

Similarly, the immune response elicited by vaccination with MVA expressing N3 (both A/Can and A/mal/NL) protected against H7N3 virus challenge, but the immune response against the heterologous N9 subtype A/Shang was not protective (Fig. 4d). Among the MVA-NA treatment cohorts, all mice vaccinated with MVA expressing N3 survived, but only 2/5 mice vaccinated with MVA expressing N9 survived and those mice lost a significant amount of body weight. This set of data suggests that MVA vectors expressing the HA or N3 of A/H7 viruses, but not vectors expressing N9, can elicit protection against the A/mal/NL virus challenge.

### Influenza-specific immune responses to MVA-HA and MVA-NA vector immunization

As described above, serum samples obtained from mice immunized with MVA vectors expressing the HA or NA of influenza A/H7 viruses induced H7-, or N3- or N9-subtype-specific IgG responses. In further characterizing the antibody response to HA and NA following vaccination with these MVA vectors, sera from vector-immunized mice were tested for strain-specific functional antibody responses. Groups of mice were vaccinated twice at an interval of three weeks with 10^8^ PFU of each HA- and NA-expressing MVA vector along with a control group vaccinated with 10^8^ PFU of MVA. Mice were bled three weeks after each immunization, the immune serum obtained from mice in each treatment group was pooled, and each serum pool tested for HA neutralizing and NA inhibiting antibodies.

The serum samples from MVA-HA vector immunized mice were evaluated in a cross-neutralization assay using lentiviral pseudoviruses expressing the HA of A/mal/NL, A/Shang, and A/Can (Table 3). Antiserum of the control MVA treatment group had a titer below the limit of detection of 30 (data not shown). A single immunization with each HA-expressing MVA elicited antibody that neutralized all three H7 pseudoviruses, with somewhat higher homologous titers in each case, confirming the cross-neutralizing response elicited by the MVA-HA vectors. A second MVA-vector immunization significantly boosted the neutralizing antibody response to all H7 pseudoviruses, suggesting the possible benefit of a prime-boost immunization strategy for poorly immunogenic or mis-matched HAs.

**Table 3:** Hemagglutinin neutralizing antibody titers in MVA-vector immunized mice.

**Immune Sera**	**Pseudovirus Neutralization (IC95)** ^**a**^		
	A/mallard/NL	A/Shanghai	A/Canada
10^8^ MVA-mallard/NL HA	**142**	128	64
2 × 10^8^ MVA-mallard/NL HA	**1233**	964	465
10^8^ MVA-Shanghai HA	58	**122**	36
2 × 10^8^ MVA-Shanghai HA	289	**387**	144
10^8^ MVA-Canada HA	80	100	**138**
2 × 10^8^ MVA-Canada HA	1193	1640	**1142**

^a^Neutralization titers were determined by neutralization of lentiviral pseudoviruses expressing influenza HA. The neutralization endpoint titer (expressed as IC_95_) was defined as the reciprocal of serum dilution causing a 95% reduction in relative luciferase units of the lentivirus expressing the H7 HA from the indicated H7 influenza virus. Homologous titers are shown in bold.

A parallel evaluation of the inhibiting antibody titer in sera from MVA-NA immunized mice was performed in a neuraminidase-inhibition (NI) assay, using homologous and heterologous viruses (Table 4). Antiserum of the control MVA treatment group had a baseline 50% NI titer of ≤10 (data not shown). As for the MVA-HA vector immunization, a single immunization with each NA-expressing vector elicited homologous NI antibody. Each N3-expressing vector also elicited cross-reactive NI antibody to the other N3 tested, but there was very little NI antibody cross-reactivity between the N3 and N9 expressing vectors. A significant increase in NI antibody titer was observed following a vector booster immunization for all groups.

**Table 4:** Neuraminidase inhibiting antibody titers in MVA-vector immunized mice.

**Immune Sera**	**Neuraminidase Inhibition (50%)** ^**a**^		
	A/mallard/NL (N3)	A/Shanghai (N9)	A/Canada (N3)
10^8^ MVA-mallard/NL NA	**640**	5	453
2 × 10^8^ MVA-mallard/NL NA	**2560**	5	1280
10^8^ MVA-Shanghai NA	5	**57**	14
2 × 10^8^ MVA-Shanghai NA	5	**640**	14
10^8^ MVA-Canada NA	226	5	**226**
2 × 10^8^ MVA-Canada NA	3620	5	**1810**

^a^NA inhibition titers were measure by ELLA as described in Materials & Methods, with MVA-N3A/mallard/NL/12/2000, H6N9 A/Shanghai/2/2016, and MVA-N3 A/Canada/rv444/2004 as sources of antigens. NA inhibition titer was defined as the reciprocal of the highest serum dilution inhibiting NA activity by ≥50%. For titer calculation, test samples with a titer <10 (below quantitation) were assigned a titer of 5. The geometric mean titers of duplicate assays of pooled serum from each treatment group are shown in the table. Homologous titers are shown in bold.

**Figure 5:**
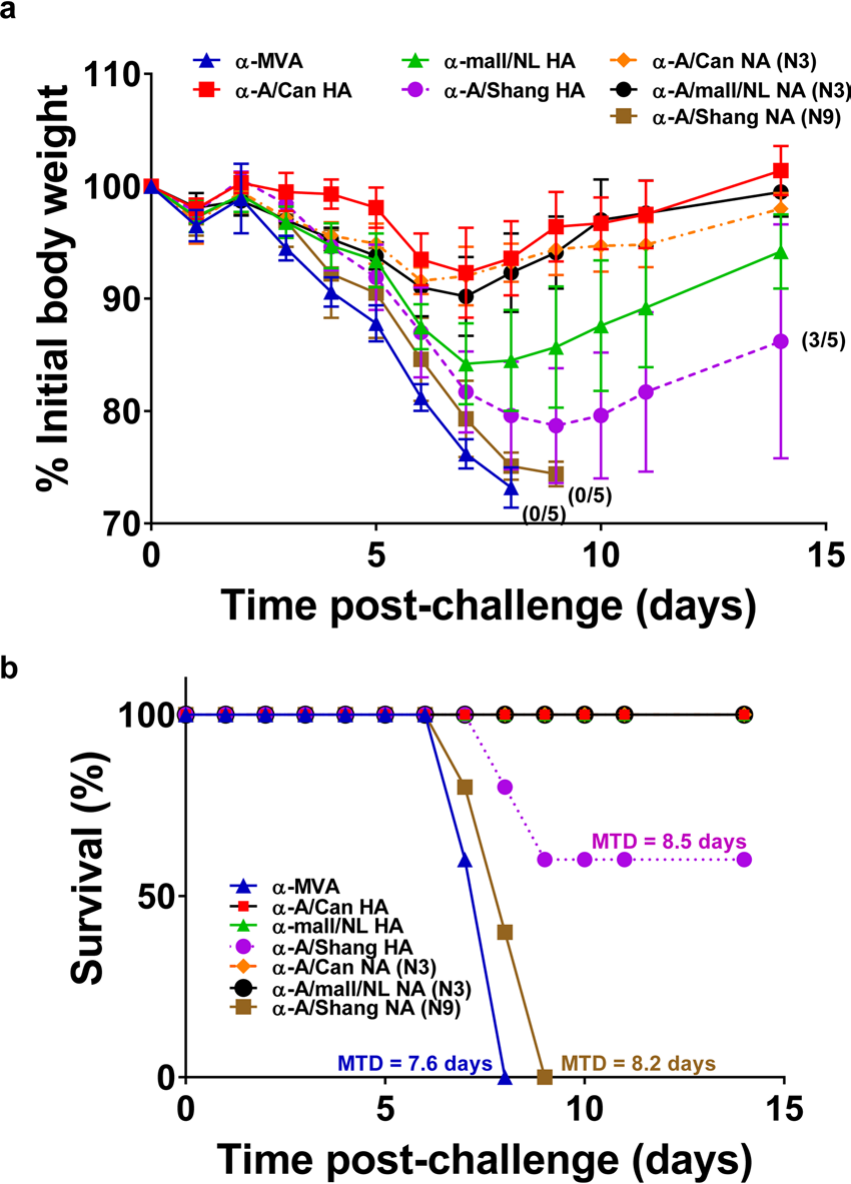
Cross-protection by passive transfer of antibody derived from MVA vector immunization. Sera was collected from mice that had been vaccinated two times with 10^8^ PFU of MVA, MVA-HA or MVA-NA vectors. Sera were administered i.p. into naïve mice (5 mice per treatment group) followed by intranasal challenge with 5 LD_50_s of A/mal/NL 6 hours post-inoculation. Mice were evaluated for two weeks and the weight change curves (**a**) and survival curves (**b**) are shown. The number of surviving mice in treatment groups with animal deaths are indicated in parentheses and the mean time to death (MTD) is shown.

In assessing the functional contribution of the antibody response elicited by MVA-HA and MVA-NA vectors to protection against H7N3 virus challenge, high-titered antisera were passively transferred into naïve mice, which were then challenged with A/mal/NL. Mice in the different treatment groups were inoculated i.p. with 0.33 mL of antiserum in a total volume of 0.5 mL, and six hours after the passive serum inoculation were challenged with A/mal/NL. Influenza morbidity in the various treatment groups in the days after challenge is shown in Fig. 5. All mice that received MVA control serum succumbed to infection (MTD = 7.6 days). All mice treated with antisera from MVA-A/Can HA, MVA-A/mal/NL HA, MVA-A/Can NA and MVA-A/mal/NL NA, survived the challenge. Three of five (3/5) of mice passively treated with MVA-A/Shang HA and none (0/5) of those inoculated with MVA-A/Shang NA (N9) survived virus challenge. Taken together, data from these studies indicate that MVA vectors expressing H7, N3, or N9 induce high titers of subtype-specific IgGs in mice. It is not clear why the homologous neutralizing antibody titer to A/Shang elicited after two immunizations with the MVA-A/Shang HA vector was lower than the homologous neutralizing titers elicited by two immunizations with the other HA-expressing vectors (Table 3), but this lower relative A/Shang antibody titer probably explains the somewhat weaker passive protection that was observed. Nevertheless, the elicited antibody responses against H7 and N3 are cross-neutralizing *in vitro* and are functional *in vivo* in providing protection against H7N3 virus challenge.

**Figure 6:**
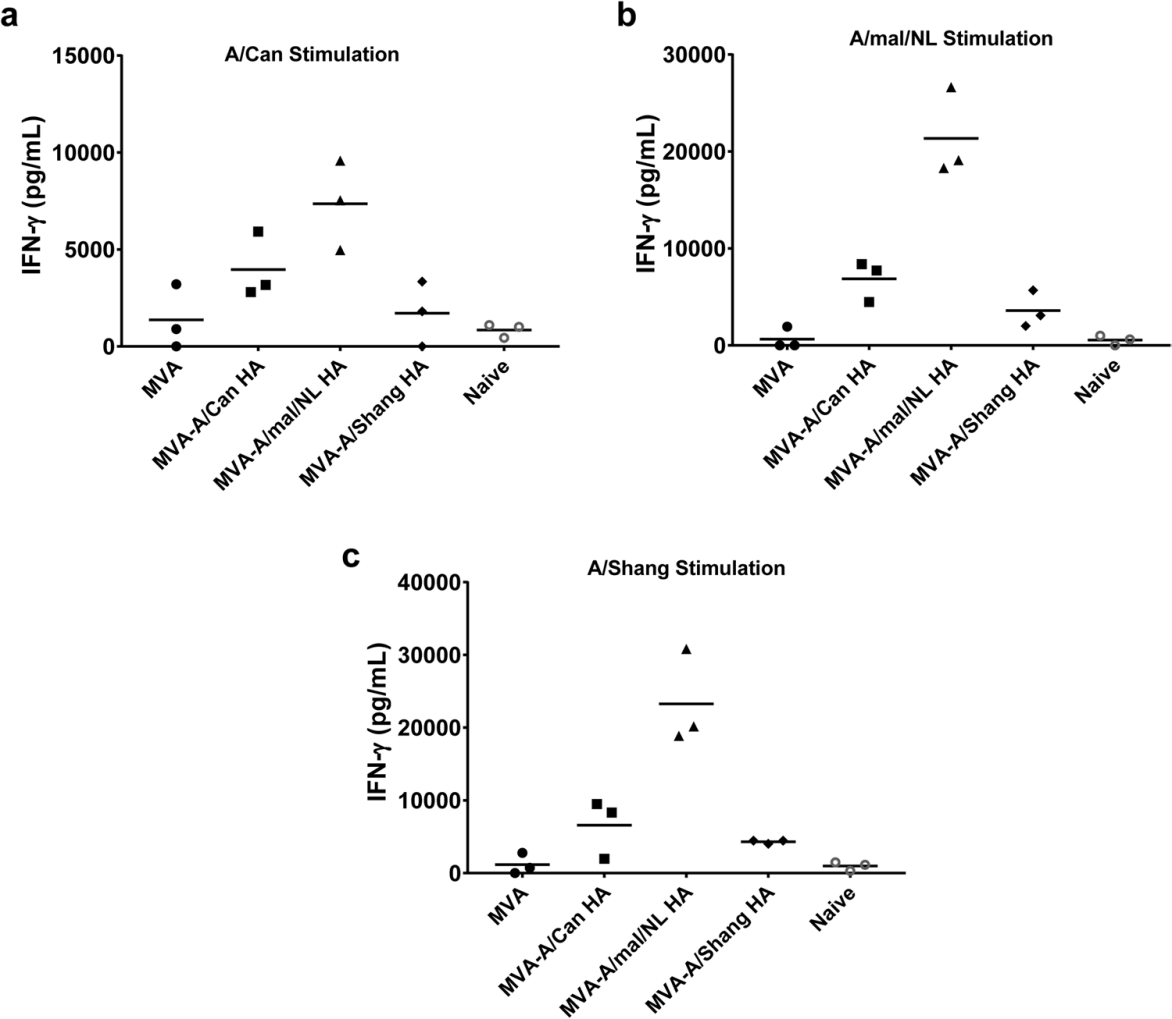
Cellular immune response to MVA-HA. Groups of mice (3 per group) were vaccinated (10^8^ PFU per mouse) with MVA-A/Can HA or MVA-A/mal/NL HA or MVA-A/Shang HA. A vector control group was vaccinated with MVA, and another control group was left untreated (naïve control). Seven days post-vaccination, all mice were euthanized and spleens harvested. Lymphocytes were cultured *in vitro* (10^6^ cells/well in 24-well plates) and re-stimulated by live virus infection (MOI of 1.0) with influenza A viruses. After 72 hrs incubation, supernatants were harvested, clarified, and tested by cytokine capture ELISA for secreted IFN-γ. The amount of secreted IFN-γ by cells re-stimulated with A/Can (**a**), A/mal/NL (**b**), and A/Shang (**c**), are shown. Each data point represents the quantity of cytokine (pg/mL) detected for each mouse minus background detected in corresponding unstimulated cells. The mean for each group is indicated by the horizontal bar.

In addition to generating an antibody response to vaccination, MVA vectors have also been shown to elicit cell-mediated immunity. Accordingly, we were also interested in determining whether the immune response induced by MVA vectors expressing H7 influenza antigens included virus-specific cellular responses. For this purpose, mice were vaccinated with 10^8^ PFU of MVA vectors expressing the HA of A/Can, A/mal/NL or A/Shang. A control group was vaccinated with MVA and a group was left untreated (naïve). Spleen cells were harvested 7 days post-vaccination, lymphocytes were cultured *in vitro* and re-stimulated by live virus infection with A/Can, A/mal/NL or A/Shang at an MOI of 1.0. Culture supernatants harvested after 72 hours of incubation were tested for the presence and quantity of secreted IFN-γ. There was no detectable IFN-γ in cells from the naïve animals and very low levels of IFN-γ in cells from the MVA and MVA-HA (Shang) animals regardless of the virus used for re-stimulation (Fig. 6a–c). On the other hand, IFN-γ was stimulated by all three influenza viruses from cells obtained from animals vaccinated with MVA-HA (Can) and (mal/NL) (Fig. 6); the IFN-γ stimulation of cells from MVA-HA (mal/NL) vaccinated animals was significantly higher than background (cells from MVA vaccinated and naïve animals) regardless of the virus used for re-stimulation. Although the number of samples evaluated in this analysis was limited, this set of data suggests that in addition to inducing robust protective antibody responses, immunization with recombinant MVA vectors expressing HA of A/H7 viruses is also capable of stimulating cellular immune responses, albeit the significance of the latter in the protection against influenza virus infection in this mouse model is not clear.

## Discussion

Several virus vector platforms have been developed as potential influenza vaccines^26^. Although still in relatively early stages of development and evaluation, these vector-based vaccines may have advantages of improved immunogenicity and production timelines compared to current types of influenza vaccines, particularly for potential pandemic influenza viruses. Considerable work in developing MVA vectors as potential vaccine vectors has been reported^2^. Such work includes several studies that have developed and evaluated MVA vectors for pandemic influenza H5 viruses, and in general, the results have confirmed the safety of H5 MVA vectors and indicated that MVA vectors expressing H5 HA induce good antibody responses that in many cases are cross-reactive to heterologous H5 sub-clades. The promising results obtained with H5 MVA vectors suggested that similar virus vector approaches might be a useful strategy to prepare for other subtypes of influenza with pandemic potential such as the novel H7N9 viruses in China in 2013, especially in light of the relatively poor antibody response observed when inactivated or subunit H7 vaccines were investigated in clinical trials^27–30^. One MVA vector expressing the HA from H7N9 A/Shanghai/2/2013 was shown to be immunogenic and protective in a ferret model of pneumonia^14^. In addition to an MVA vector approach, other viral vector vaccines for protection against H7 viruses have been reported^31,32^ as well as live attenuated H7 vaccines^33,34^. All of the H7 virus vectors and inactivated H7 vaccines to date have focused on the immunogenicity and protective effect of H7 HA.

In the study reported here, we set-up a mouse challenge model with an H7N3 virus and compared the protective effect of MVA vectors expressing the homologous HA and NA from this virus, as well as the HA and NA from another H7N3 virus from a North American H7 lineage and the HA and NA from an H7N9 virus representative of the first wave of H7N9 viruses that emerged in China in 2013. Similar to previously reported studies, an MVA vector expressing the homologous HA was protective over a wide range of doses. However, in addition we found that MVA vectors expressing heterologous H7 HAs were protective, demonstrating the cross-protective potential of a non-matched MVA H7 vector vaccine. At the global amino acid level, the A/Can and A/Shang HA are 86.8% and 97.1% identical to the A/mallard HA, respectively, and the antibody response to these MVA expressed heterologous HAs was sufficiently broad to cross-neutralize pseudoviruses bearing HA *in vitro* and provide protection when passively administered before A/mallard H7N3 challenge. It is not known how the antibody response to an MVA vector-expressed HA compares with that of a traditional inactivated vaccine, but at least in studies of H5N1 inactivated vaccines, adjuvants have usually been needed to generate a broader antibody response^35^.

Very few studies using MVA vectors to express influenza NA have been reported. In one study, an MVA vector expressing the H1N1pdm2009 NA was only partially protective against a homologous influenza virus challenge^36^. In our experiments, an MVA vector expressing the homologous NA was as protective against lethality as the HA vector over a similar range of doses. However, mice that were vaccinated with 10^4^ or 10^5^ PFU MVA-HA lost less weight than those vaccinated with equivalent PFU of MVA-NA, suggesting that the protective response to the MVA H7 might be somewhat more effective than the protective response to MVA N3 against H7N3 influenza challenge. The MVA vector expressing a heterologous N3 (92.5% amino acid identity) was also protective, but the MVA vector expressing the N9 from the H7N9 virus (45.0% identity) was not. Since antibody to HA and NA have been shown to be independent correlates of protection^37^, it was somewhat surprising that co-administration of MVA-HA and MVA-NA did not result in obvious improved protection in our challenge experiments. This may be due to limitations in the challenge model used. For example, at doses of MVA vector of 10^4^ or greater, all mice responded with detectable HA-specific antibody and all were protected from challenge. At the lower immunization doses used for the co-administration experiments, antibody responses were not evident in all animals, suggesting that there might be a “take” threshold that must be reached for successful immunization. Additional measurements of pathogenesis such as measuring the viral load in the lungs might add additional information. But, it is also possible that a challenge model that employs higher immunization and challenge doses might be able to demonstrate an additive effect of HA and NA MVA vector immunization.

Since the antibody response to influenza virus infection is known to be critical for effective control of influenza disease, and since most influenza vaccines contain HA as the vaccine antigen, most of our analyses of the immune response to MVA vector immunization focused on measuring and understanding the antibody response to HA and NA. Nevertheless, cell-mediated immunity is known to be an important component of the immune response to influenza virus infection, and indeed, other MVA vectors designed to elicit cell-mediated immunity to conserved proteins such as NP have been described, including some that have been evaluated for protection against H7 virus challenge^38^. In our limited analysis, immunization with recombinant MVA vectors expressing HA of A/H7 viruses also appeared capable of stimulating cellular immune responses, but the role of cell-mediated immunity to HA and NA in the context of a robust protective antibody response to those proteins is difficult to determine. Additional immunization approaches employing MVA vectors expressing H7N9 HA and NA, along with vectors expressing conserved proteins, could be explored as a means to maximize a broad protective response to H7 viruses.

The results from the current study add support to the concept of an MVA vector platform as a reasonable and feasible vaccine strategy for pandemic influenza. It has been suggested that repositories of MVA vectors be established for potential pandemic influenza strains^1^, and the MVA vector-mediated cross-protection demonstrated in our study, as well as other studies, seems to indicate that an MVA HA or NA-expressing vector might not need to be perfectly matched to an emerging virus strain in order to provide protection. The promising results with MVA NA vectors provide another option for an MVA vector repository since the drift of HA and NA does not occur at the same rate. Future steps in the development of MVA vectors as a potential pandemic influenza response will include a more complete characterization of the protective response to vaccination, including a finer analysis of both antibody and cell-mediated immune responses, and a more thorough evaluation of the broadness of the protective response. In addition, studies that compare the immunity and protection afforded by MVA vectors to that elicited by other types of influenza vaccines, particularly traditional inactivated vaccines, will be important. Further, additional studies using MVA vectors expressing HA and NA glycoproteins and conserved viral proteins, as well as prime-boost studies that evaluate a mismatched vector or vaccine followed by a matched vaccine, will be important for developing optimal immunizations strategies. Finally, to be truly feasible as a pandemic response, licensure of an MVA vector vaccine should be pursued in advance of the emergence of a novel influenza virus.

## Electronic supplementary material

Supplementary Info File for Fig 1
