# Supplementary material for: Immunogenicity and Protection Against Influenza H7N3 in Mice by Modified Vaccinia Virus Ankara Vectors Expressing Influenza Virus Hemagglutinin or Neuraminidase
Source: Sci Rep. 2018 Mar 29;8:5364. doi: 10.1038/s41598-018-23712-9 (PMC5876369; doi:10.1038/s41598-018-23712-9)
Supplement: Supplementary file 1 — Supplementary Info File for Fig 1 [file 41598_2018_23712_MOESM1_ESM.pdf]

Supplementary Information for

"Immunogenicity and Protection Against Influenza H7N3 in Mice by Modified Vaccinia Virus Ankara Vectors Expressing Influenza Virus Hemagglutinin or Neuraminidase"

Clement A. Meseda[1], Vajini Atukorale[1], Jackeline Soto[1,] Maryna C. Eichelberger[2], Jin Gao[2], Wei Wang[3], Carol D. Weiss[3], and Jerry P. Weir[1]

[1]Laboratory of DNA Viruses, [2]Laboratory of Respiratory Viral Diseases, [3]Laboratory of Immunoregulation, Division of Viral Products,
Center for Biologics Evaluations and Research, Food and Drug Administration
10903 New Hampshire Ave, Silver Spring, MD 20993

Full-length gels used in Figure 1.

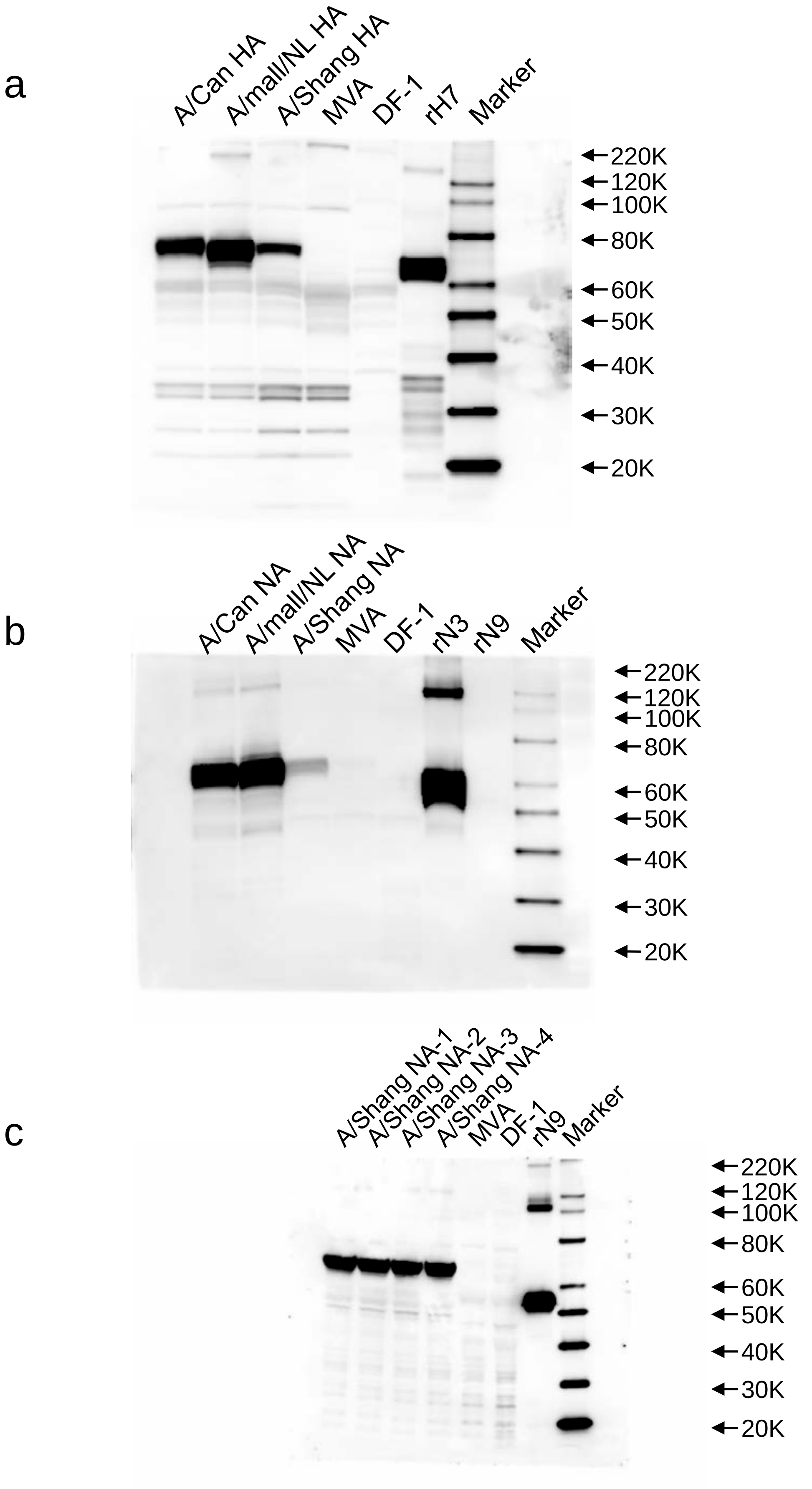

Figure S1. Western blot analysis of recombinant MVA vectors expressing influenza HA or NA. The full Western blot images for Figure 1 (a, b and c) are shown.
